# Viscoelastic Response of Neurofilaments: An Atomistic Simulation Approach

**DOI:** 10.3390/biom11040540

**Published:** 2021-04-07

**Authors:** Md Ishak Khan, Fuad Hasan, Khandakar Abu Hasan Al Mahmud, Ashfaq Adnan

**Affiliations:** Department of Mechanical and Aerospace Engineering, University of Texas at Arlington, Arlington, TX 76019, USA; mdishak.khan@mavs.uta.edu (M.I.K.); fuad.hasan@mavs.uta.edu (F.H.); mahmud.khandakarabuhas@mavs.uta.edu (K.A.H.A.M.)

**Keywords:** neurofilaments, axonal cytoskeleton, axonal injury, mechanical behavior, viscoelastic modeling

## Abstract

Existent literature has limitations regarding the mechanical behavior of axonal cytoskeletal components in a high strain rate scenario, which is mainly due to limitations regarding the structure of some components such as tau protein and neurofilaments (NF). This study performs molecular dynamics (MD) simulations on NFs to extract their strain rate-dependent behavior. It is found that they are highly stretchable and show multiple stages of unfolding. Furthermore, NFs show high tensile stiffness. Also, viscoelastic modeling shows that they correspond to simplified viscoelastic models. This study effectively enhances the existent axonal models focusing on axonal injury.

## 1. Introduction

Axonal cytoskeleton is primarily composed of neurofilaments (NF), microfilaments (MF), and microtubules (MF) crosslinked by MT-associated proteins (mainly tau). Among these, the mechanical behavior of MFs and MTs has been studied in depth, and of NFs has been studied partially in the past. However, mechanical behavior insight of NFs, especially in a high strain rate scenario relevant to TBI, is not present in the literature. NFs build up axonal cytoskeletons in neurons by cooperating with microtubules (MT). NFs consist of five types of proteins: neurofilament light (NF-L), neurofilament medium (NF-M), neurofilament heavy (NF-H), internexin, and peripherin [1,2,3]. The co-assembly of these five types of proteins can be different according to stages of development and nerve cell types. NF expression is directly related to development, as it increases postnatally in neurons that are myelinated [4]. Aside from their structural role, they can act as cargoes of axonal transport [1]. NF is also important as a biomarker of axonal damage in neurodegenerative diseases, because during axon degeneration, NFs are released into the blood or cerebrospinal fluid (CSF) [5].

Brain NFs are particularly different from the NFs of the other parts of the body, because they are prone to create a more viscous mixture with MTs (viscosity differing around 1000 cP), and microtubule associated proteins (MAPs) act as cross bridges between these two types of proteins, as suggested by the affinity grid electron microscopy study on bovine brains [6]. Accumulation of NFs is a distinctive biomarker of neurological damage or disorders [7,8], such as frontotemporal dementia or multiple sclerosis, as suggested by multiple studies [9,10]. Presence of soluble NFs in cerebrospinal fluid (CSF) or increased NF level have been related to neuronal death or axonal degeneration [11], and stoichiometry of NF types differs according to the type of pathology [12]. Furthermore, NF accumulation in different phosphorylated states is also related to several pathologies [13].

**Figure 1:**
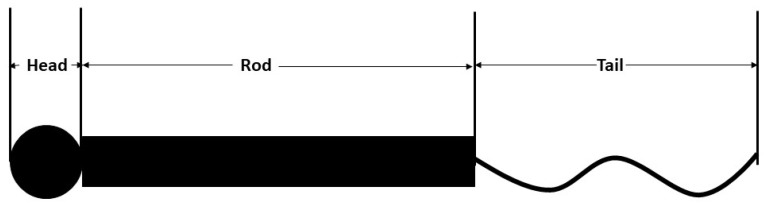
NF structure depicting the head, rod, and tail domains. The head and rod domains are almost the same for all isoforms, while the length of the tail domain is variable, increasing in length from light isoform (NF-L) to heavy isoform (NF-M and NF-H). For details, the reader may refer to the work of Jayanthi [30]. The image is solely for obtaining an idea about different domains of NF and is not drawn to scale.

There have been several hypotheses proposed to elaborate NF structure and behavior including review works [4,14], as NFs contain domains with intrinsic disorder. The structure of an NF contains central alpha-helical region flanked by unstructured N and C termini. The general description suggests that the structure contains a globular head (N terminus); a hydrophobic alpha-helical rod domain [15] and variable; and an intrinsically disordered, tail domain (C terminus) differing in length and amino acid composition (Figure 1) [16]. The N terminal head domain contains an MT polymerization inhibitory domain that regulates the number of MTs in the axon and facilitates forming end-to-end interaction of heterotrimers to form complete filaments [17], and the rod domains polymerize NF subunits while simultaneously working as a binding site for the myosin [18]. Finally, the C-terminal domains of NFH and NFM form fine lateral extensions that create spacing between NFs, maximizing the space-occupying capability during axon caliber expansion [19]. Conformational properties of interacting NF structure, charge states of the sidearms, etc. have been analyzed in depth in separate studies [20,21]. The substructures have also been studied in separate research earlier [22], and it is found that the equilibrium structure is determined by ionic strength and pH. Moreover, the electrostatic interactions between the charged portions of the structures play a large role in the formation of NF network [23]. Sequence-based modeling has revealed NF sidearm structure in earlier studies [24], and suggested that medium, not extensive, protrusions are critical to define NF spacings and eventually, axonal caliber. In other words, NFM defines the axonal diameter, because even at phosphorylated state, NFHs get stretched but do not unfold fully, staying in the bounds of the NFM sidearms [24]. This observation is in conflict with several studies that suggested that NFH has the dominant role in regards to axonal diameter [25,26], while agreeing with some experimental results [27,28]. However, the role of NFM phosphorylation should not be overemphasized, because gene replacement technique that produces phosphorylation incompetent alanine showed that there is no significant difference between wild type and phosphorylation incompetent sidearms [29].

There have been several studies to model NF network and interaction, such as sequence-based coarse grained (CG) modeling where phosphorylated state was controlled by assigning appropriate charge to KSP motifs [24]. Monte Carlo simulation studies [12,17] strengthen the polymer brush appearance of the NF structure, and that phosphorylation state controls the stretching, interaction, and conformation of the sidearms. Conformational study performed by molecular dynamics (MD) simulation agrees with the general hypothesis that NF is a polyelectrolyte (NFL is a strong one, while NFM and NFH are weaker) [21].

One of the most highlighted aspects of NFs is the charged nature of the tail domain (the sidearms differ in protein length, net charge and charge distribution [24,31,32]). Although the tail region is mostly negatively charged, there are residues containing positive charge in this region as well. The self-assembly occurs due to the coiled coil interactions along the hydrophobic strips of the alpha-helical region [15]. Electrostatic analysis has shown that due to the polyampholyte nature of the sidearms, there is a handshake (attractive or repulsive) interaction mechanism between the regions of separate NF subunits (tip-tip or tip-body). Additionally, the tail region consists of a nonuniform charge distribution due to amino acid residues that depend on pH control (due to ionizable groups), as well as the level and distribution of phosphorylation sites [24]. The effects of divalent ions, which can act as effective crosslinkers between adjacent filaments, have been emphasized in some studies, and it is hypothesized that multivalent ions screen the repulsive interactions between charged residues on the sidearms leading to their collapsed conformation, and induced a cross-bridge type of interaction between the adjacent filaments [33]. Furthermore, hydrophobic region interactions between adjacent filaments have also been found to reduce the extension of the sidearms [30]. Among other parameters, one worth mentioning is exposure to neurotoxic aluminum, which induces perikaryal accumulation of phosphorylated NFs by stabilizing cross bridging of the sidearms [34]. Another one is the proportion of occurrence of NFM and NFH, to which the network has been found to be remarkably stable by self-consistent field theory [35].

Mechanical properties of NF networks have been found to be quite similar to those of semiflexible polymers [36], the storage modulus of which is highly concentration dependent. The storage modulus increases significantly with aluminum ion (the ion which also incorporates brittleness to NFs). Bovine spinal cord NF study through fluorescence and electron microscopy revealed the properties of the viscous gel formed by NFs (elastic modulus >100 Pa and shear moduli increased in a time-dependent manner to the level of vimentin i.e., >100 Pa, suggesting the importance of the cross-bridges) [37], which suggested that phosphorylation, dephosphorylation, and interaction with other cytoskeletal components (such as actin) are related to the mechanical properties. Nonlinear elasticity has been found in tensile and shear test on intermediate filaments (IF) networks, along with strain stiffening and recovery [38]. Linear and nonlinear viscoelastic studies showed that NF network works as a soft solid and exhibits significant strain stiffening above critical strain (30–70%) [39]. The elasticity is entropic in nature and can be related with cross-linked semiflexible network, mediated by divalent ions.

There are several unanswered questions or areas regarding NFs, such as specification of the regions regulating transport kinetics, mechanics of formation, and stabilization of stationary NF network affected by phosphorylation, etc. which require further studies to obtain conclusive insight. In the current study, an attempt has been made to determine the stiffness of NF isoforms at different strain rates. In principle, such a study calls for deeper understanding of the viscoelastic properties of NFs. From a broader perspective, this study contributes to existent axonal modeling [40,41] and provides new insight regarding high strain rate behavior of axonal cytoskeletal components; as such, insight is required for the improvement of research performed focusing on traumatic brain injury (TBI) scenario [42,43,44,45].

Viscoelastic modeling of a material consists of fitting the stress–strain response of that material to an established constitutive equation. There have been detailed texts that rigorously discuss the methodologies of viscoelasticity for materials [46]. The viscoelastic response can be obtained by performing familiar approaches of creep or relaxation tests–according to the nature of the material. If the applied strain causes a time-dependent stress, then the modulus is also a function of time, and thus we calculate the relaxation modulus for the respective material. The deformation mechanism, however, depends on the characteristics of the material. Determination of viscoelasticity is not just limited to performing relaxation and/or creep tests, but also determining the linearity of the material, which is determined by developing isochronous diagrams, i.e., if the relaxation modulus (or creep compliance) is only a function of time and not a function of stress level, then the material is defined as a linear viscoelastic material, otherwise it is called a nonlinear viscoelastic material. After determining the viscoelastic response, the rest of the modeling consists of fitting the relaxation or creep response to established phenomenological viscoelastic models, such as Kelvin-Voigt or Maxwell models. Without going into details of theoretical discussions on such models, we can mention the feature of Kelvin or Maxwell models that they can increase the parameters by series or parallel addition of the spring or dashpot elements in the constitutive equation to satisfy our material behavior, which leads to a generalized Kelvin or Maxwell model. Their expressions are, in various occasions, convenient to study by expressing them with Prony series expression. Prony series development based on strain rate response have been studied in several independent works and paved the pathways for further analysis [47,48]. In biomolecules, the viscoelastic nature of them has been hypothesized to control their dynamics. For example, viscoelasticity of tau protein, which is an microtubule (MT) associated protein and acts as a crosslink between MT fibers, has been found to dictate the reversible sliding and failure of MT, as suggested by experimental and computational works [40,41,49,50]. However, viscoelasticity of NFs has not yet been characterized in an organized manner, except some experimental insights obtained from NF-MT mixture studies, and their tendency to form viscous gel, as suggested by electron micrography and other experimental studies [33,39,51,52,53].

The shortcoming in this regard is evident, and therefore, it is necessary to perform viscoelastic modeling for NFs to provide required insight into complete axon modeling, which incorporates component-level response. This study has undertaken a simplified approach to model stress–strain response of neurofilaments (NFs) by performing relaxation tests on them after stretching them at different strain rates, and then fitting them to the linear viscoelastic model. This study is therefore important to obtain insight into the viscoelastic response of a key cytoskeletal component, which will facilitate the development of a computational model for an axon. In other words, this study attempts to address the key limitation in the literature, which suggests that there is a lack of insight in mechanical behavior of tau protein and NFs [54]. The tensile tests are performed by MD simulation due to their earlier successful implementation in extracting high strain rate response of axonal cytoskeletal components. The stress vs. strain data from the tensile tests are then utilized to fit them with viscoelastic models. Therefore, this study provides significant insight regarding mechanical behavior of NF isoforms.

## 2. Method

### 2.1. MD Simulation Scheme

In TBI scenario, the smallest representative element that can depict the deformation of axon contains MT, a dimerized tau protein, NF, and actin. As mentioned in the conclusion, the long-term goal of this modeling study is to build a realistic bottom-up axon model. To achieve this, filament-level behavior of the constituents is needed to be determined, which is the objective of this study (in our earlier work, we have determined high strain rate response of tau protein [55]). Furthermore, one of the purposes of the sidearm (tail) region is to create spacing between NFs, as mentioned in the introduction. Therefore, it can be assumed that in the smallest representative model, using only one NF is adequate. As mentioned in the introduction, the head domain of NFs plays a role in MT polymerization, and the rod domain contributes to NF polymerization. As the representative model in this study contains only one NF, and the objective is to extract the mechanical response of the tail region, mechanically fixing the head and rod domains is a viable approach.

To determine the mechanical behavior of NF at a high strain rate—the insight regarding what is absent in current literature as indicated by a very recent study [55]—the tensile tests are performed in two phases. The objective of the first phase is solely to extract the unfolding and stretching mechanism and dependence on strain rate. The second phase, however, is focused on performing a tensile test for a significant stretch and then fitting the afterward relaxation stress versus strain response to the simplified viscoelastic model. To facilitate this, the methodology is designed as below:

The structures of NFs (both NF-L and NF-H) are obtained from i-TASSER [56]. Recent studies have substantiated that it is acceptable to proceed with the predicted structure for proteins consisting of a disordered portion in the filament [55,57,58,59].

It is necessary to solvate the protein structures in water molecules. Therefore, reasonable sizes of simulation boxes are created for each protein. After creating the simulation box, CHARMM-GUI modules are used to solvate them with TIP-3P water molecules [60]. For explicit water molecules being used as solvents, CHARMM-GUI facilitates adding ann appropriate number of neutralizing ions, so that the system can attain electrostatic stability easily. For this study, the required number of 0.15 KCl ions are added for neutralization. The force field is essentially CHARMM, and as the simulation running platform is LAMMPS, the lj/charm/coul/long module is used to define the coulombic long-range interactions [61,62]. Appropriate CMAP corrections are used for CHARMM force-field, which is a norm for protein systems [61,63]. Moreover, pppm solver is used for the interaction calculation, and the inner and outer cutoff radii used are 10 Å and 12 Å, respectively. The simulation box sizes, number of atoms in the simulation box, and approximate filament length are provided in Appendix A and Appendix B.

Before performing tensile tests, the systems are equilibrated for 1 ns (310 K, NVT ensemble). The sufficient equilibration is ensured by using energy minimization scheme before equilibration (min_style cg) and observing potential energy vs. time graph throughout the process.

After the appropriate extent of equilibration, the tensile tests are performed in two phases. The objectives are mentioned at the beginning of the method section. For the first set of simulations, NF filaments are subjected to tension. In the TBI scenario, the likely concerns are unfolding and stretching of NF sidearms. Therefore, the head and rod domains of NFs are kept fixed, while the sidearms are pulled. The applied strain rates are moderately high to very high, and relevant to TBI scenario (10^8^ s^−1^ to 10^9^ s^−1^), which is realized during cavitation bubble collapse phenomena taking place in blast incidents. The stress and strain data are extracted as described in the following paragraphs.

The strain data are extracted from the displacement of the atoms, which is convenient in LAMMPS. However, for stress data, per atomic stress calculation is implemented. Due to the stress data being associated with the volume of the group of atoms, Voronoi cell volume approximation is used to extract stress data from the per atomic stress output (the stress data are generally produced in pressure multiplied by volume format, and therefore, the Voronoi cell approximation is used to divide the generated stress data by the approximate volume, so that the stress data can be extracted). This feature is only available through the voro++ feature of LAMMPS [64].

For the strain rate dependence, NF filaments are stretched to an extent so that both unfolding and stretching are realized. However, for viscoelastic modeling, 80% stretch is performed, and then the responses are fitted with viscoelastic models. For this part, cvel command (which is a part of LAMMPS steered molecular dynamics or smd scheme) is implemented.

### 2.2. Viscoelastic Modeling of NF

The simplified viscoelastic modeling scheme needs to be elaborated here. We know that the simplified expression for one-dimensional relaxation test is:

(1) $$E\left(t\right)=E_{\infty }+\sum_{i=1}^{N}E_{i}e^{\frac{-t}{\tau_{i}}}$$

where E(t) = relaxation modulus, E_∞_ = long-term modulus, t = time, and τ = relaxation time. Now, if we replace the long-term modulus by E_1_ (solely for convenience, it has no relation to popularly used terms in elastic theory), and then denote the rest of the expression of E by E_2_, then the simplest expression will be,

(2) $$E\left(t\right)=E_{1}+E_{2}e^{\frac{-t}{\tau }}$$

where E values are in GPa, and τ is in ps. This expression is the one that we are calling as a simplified three-parameter model (the parameters being E_1_, E_2_, and τ). In case the fitting shows that the long-term modulus (E_1_) is zero, we call it a simplified two-parameter model. Now, for fitting with the three-parameter viscoelastic model, we have tracked the time-dependent elastic modulus, E(t), for each filament, and used it in a simplified MATLAB code to fit to the above simplified three-parameter model, using three guessed values of the constitutive equation. The error in the fitting is minimized by using *fminsearch* function of MATLAB [65], which minimizes the norm of the function generated by the difference of E(t) vs. time graph and three parameter function graph obtained from the guessed values.

Now, it is sufficient to fit the E(t) vs. t graph obtained from the relaxation data to estimated E_1_, E_2_ and τ values. It is to be mentioned that as per our need, we can extend the equation to fit the result with the simplified model with more parameters (5, 7, and so on). However, in our case, three-parameter fitting was sufficient.

After obtaining the fitted E(t) expression, we can use Alfrey’s elastic-viscoelastic correspondence principle to obtain time-dependent shear modulus, G(t), and time-dependent bulk modulus, K(t) for each case, by using these steps briefly stated here [46]:

(a) Obtain E(t),
(b) Obtain E(s)*, which is the expression of E at the Laplacian domain,
(c) Calculate G(s)* and K(s)* using Equations (3) and (4) (these are the expression of G and K at the Laplacian domain, respectively),
(d) Use inverse Laplace calculation to obtain G(t) and K(t).

As mentioned at step b, the conversion equations for G and K are shown in Equations (3) and (4).

(3) $$\bar{G{\left(s\right)}^{*}}=\frac{\bar{E{\left(s\right)}^{*}}}{2(1+\bar{\nu {\left(s\right)}^{*})}}$$

(4) $$\bar{K{\left(s\right)}^{*}}=\frac{\bar{E{\left(s\right)}^{*}}}{3(1-2\bar{\nu {\left(s\right)}^{*})}}$$

As we know the expression of G and K using the E in the Laplacian domain, we can easily convert them to time domain expressions by using inverse Laplace calculation. Finally, we obtain the relaxation modulus, relaxation shear modulus, and relaxation bulk modulus for the particular filament at that particular strain rate. The final expressions for G(t) and K(t) are shown in Equations (5) and (6).

(5) $$G\left(t\right)=G_{1}+G_{2}e^{\frac{-t}{\tau }}$$

(6) $$K\left(t\right)=K_{1}+K_{2}e^{\frac{-t}{\tau }}$$

However, the conversion in Equations (3) and (4) requires a Poisson’s ratio, and therefore, we have used two scenarios in our calculation: one is for normal, compressible material assumption (Poisson’s ratio, ν = 0.3), and another is for nearly incompressible material assumption (Poisson’s ratio, ν = 0.48). Currently we lack the Poisson’s ratio calculation in the existent literature, therefore, we had to approach with these two assumed values. However, from microrheological measurements of MT-Actin network study [66] and orthotropic elastic shell model for MTs [67], we have found that the Poisson’s ratio of such axonal cytoskeletal components is between 0.3–0.4. Therefore, our assumption is that the Poisson’s ratios we have used in the calculation are reasonable for NFs.

It is to be noted that the simulation snapshots that are shown in the later section of the manuscript are generated via OVITO [68]. All the simulations are performed by Stampede 2 supercomputer of Texas Advanced Computing Center (TACC).

To clearly describe the work sequence for the simulation scheme, the following list is provided:

- Determining the NF-L and NF-H structures from i-TASSER (pdb files),
- Using CHARMM-GUI (quick MD simulator or solution builder module) for creating the simulation box, solvating the system along with neutralization by the necessary number of ions, and creating LAMMPS readable data file for simulation,
- Creating input scripts to run simulation in LAMMPS (equilibration and tensile test),
- Running equilibration for energy minimization in LAMMPS,
- Running tensile test in LAMMPS,
- Post-processing (calculating stress and strain from the log file, plotting the data in MATLAB, fitting the stress vs. strain data to required expression by using *fminsearch* function of MATLAB, creating snapshots of the simulation by using OVITO modifiers).

## 3. Results

### 3.1. Neurofilament Deformation

**Figure 2:**
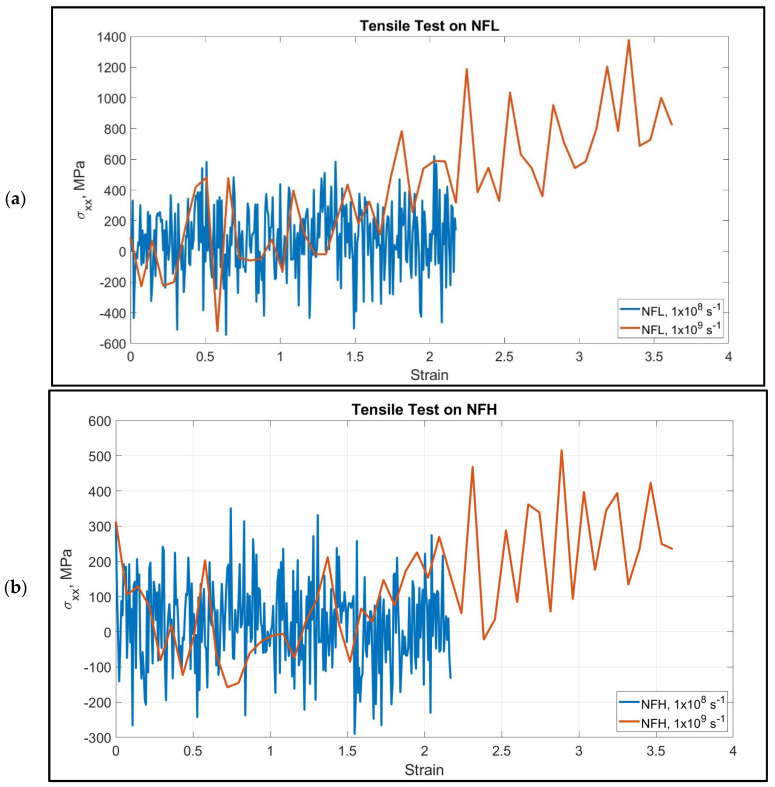
Stress–strain graphs at two different strain rates for the pulled tail domain of (**a**) NFL and (**b**) NFH. Throughout the pulling process, we can observe simultaneous unfolding and stretching, and therefore, these two domains cannot be distinguished. However, NF acts as a stiffer material under the application of higher stress as depicted by the steeper slope at higher strain rate. In order to make a reasonable compromise with the computational cost, we have stopped at ~220% strain for lower strain rate, which we assumed to be sufficient to capture stretched state, while for higher strain rate, we have recorded result till ~360% strain.

We have performed tensile tests at high strain rates on the NF isoforms (1 × 10^8^ s^−1^ and 1 × 10^9^ s^−1^). The head and rod domain atoms were fixed, and the last few atoms of the tail domain were pulled towards −x direction. The calculated stress–strain graphs are shown in Figure 2. We have assumed that >200% strain of the tail domain is sufficient for determining their unfolding and stretching stiffness. NF tail showed unfolding to a significant extent before going into the stretching region.

The stretching and unfolding, however, are observed simultaneously. The last portion of the tail was under pure stretching, while the portion attached to the rod domain was still unfolding.

**Figure 3:**
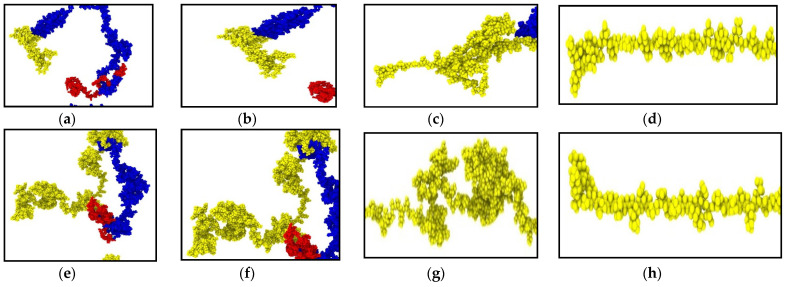
Snapshots at different timesteps during the tensile test performed on NFL and NFH (strain rate: 1 × 10^9^ s^−1^). (**a**) Initial structure of NFL; (**b**) NFL tail region: 0% strain; (**c**) NFL tail region: 180% strain, showing simultaneous unfolding and stretching; (**d**) NFL tail region: 360% strain, showing pure stretching at significant portion of tail region; (**e**) Initial structure of NFH; (**f**) NFH tail region: 0% strain; (**g**) NFH tail region: 160% strain, showing simultaneous unfolding and stretching; (**h**) NFH tail region: 320% strain, showing pure stretching at significant portion of tail region. Color legend: Red: head domain, blue: rod domain, yellow: tail region.

However, the consistency we have found in their behavior is the dependency of the stiffness on the strain rate–that is, NF behaved as a stiffer material under the application of higher strain rate, and it can be ~0.5 GPa. Another important aspect is the filament length, which, to our observation, plays an important role to determine the stiffness. Under the application of the same strain rate, the smaller filaments showed higher stiffness than the longer filaments, which is an expected result. Most of the isoforms showed steady increase over the range of 0–217% strain. In NF-H under both strain rates, the stress–strain response was not steady until they unfolded to 50–150% strain. This behavior can be attributed to the longer filament length, as for longer filament, the development of tensile stress requires more unfolding to be considered as a significant amount. It is to be considered that the head and rod domains of the NF structure are conserved, while the tail is the variable part (lengthwise) of the isoforms due to the inherent disorder. In NF-L, the tail portion is comparable in length to the rest of the structure, while in NF-M, it is significantly longer, and in NF-L, it is a few times longer than the rest of the structure, and therefore, bound to show deviation from the behavior observed in case of NF-L. Figure 3 shows different stages of unfolding and stretching of the NF isoforms under different strain rates.

### 3.2. Viscoelastic Modeling of Neurofilaments

**Figure 4:**
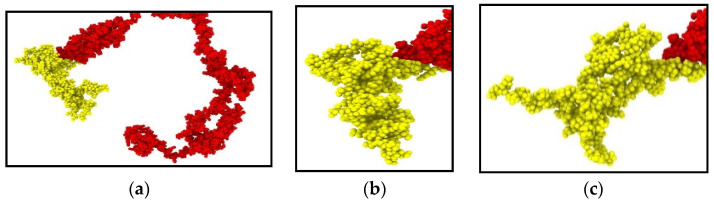
Pre-relaxation stretching for NFL (1 × 10^8^/s). (**a**) Initial structure (0% strain), (**b**) sidearm region at mid-stretch (40% strain), (**c**) sidearm region at end of stretch (80% strain). Color legend: Red: head and rod domain, Yellow: sidearm.

**Figure 5:**
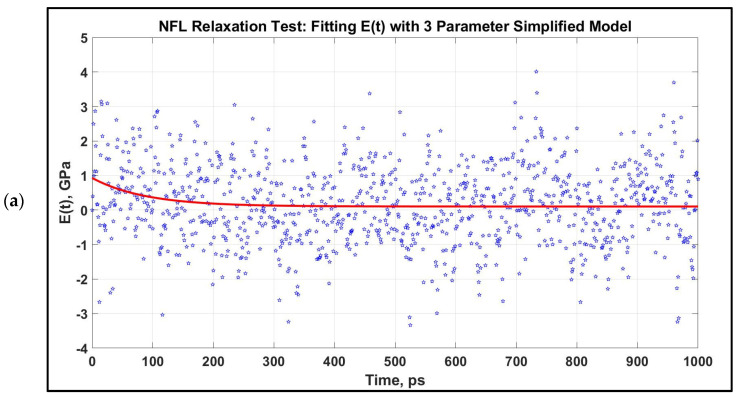

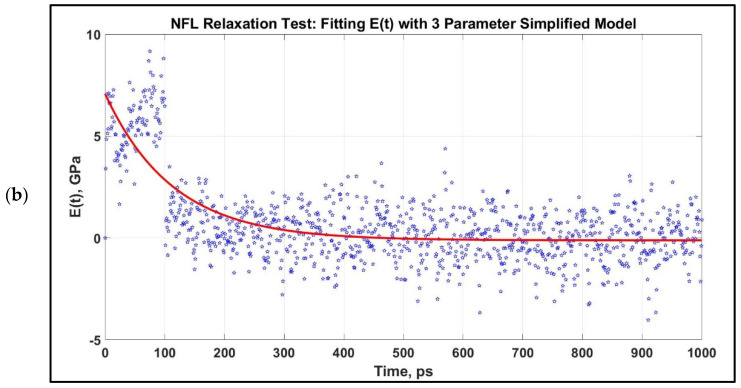
Modulus, E(t) vs. time for 1 ns relaxation test for NFL, which are stretched to 80% at two strain rates. The relaxation curves at two strain rates are not significantly distinct for NFL. After obtaining the E(t) vs. time graph, they are fitted to a three-parameter simplified model for an initial stretch of 0.8 at two different strain rates. (**a**): 1 × 10^8^ s^−1^, (**b**): 1 × 10^9^ s^−1^.

**Figure 6:**
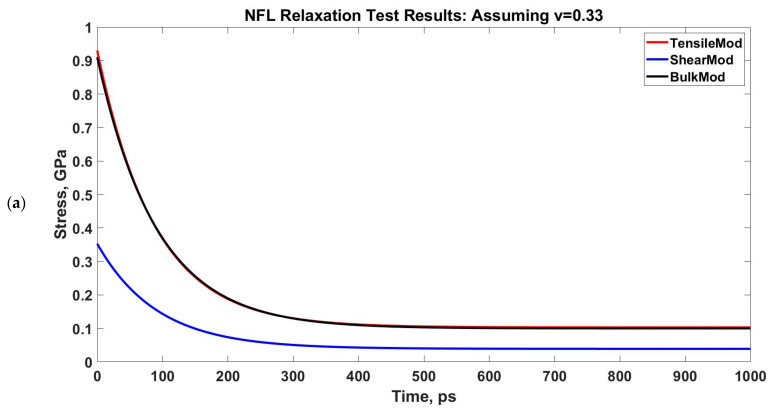

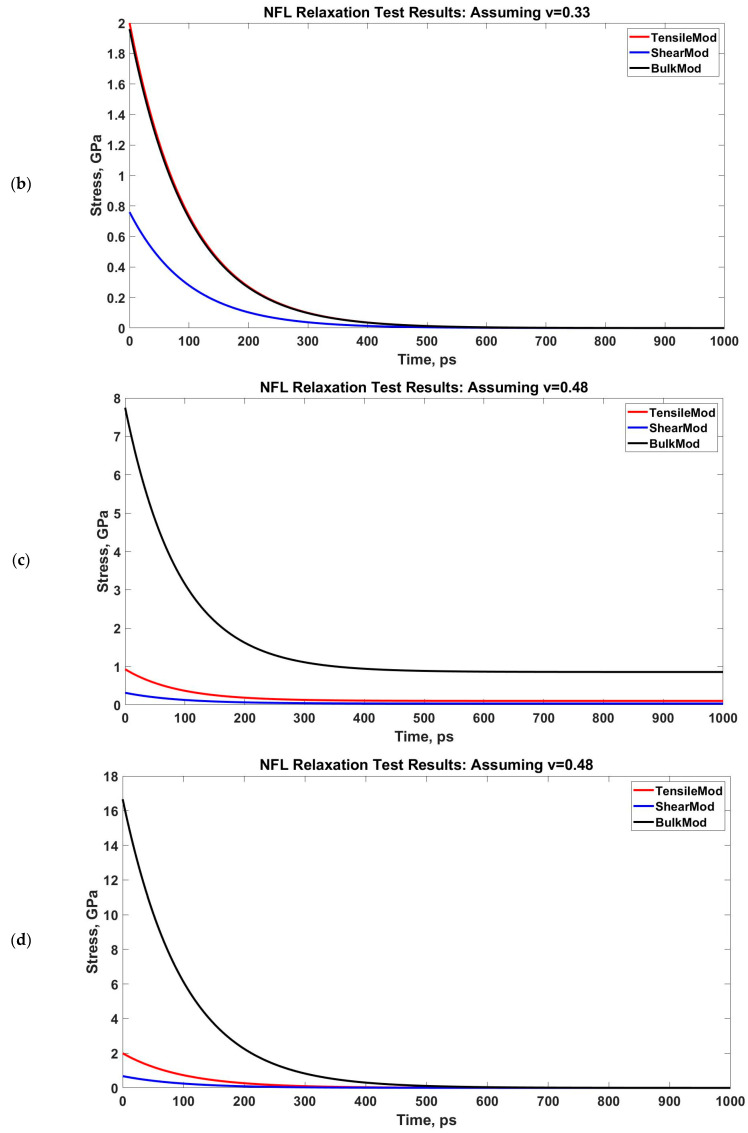
Determining G(t) and K(t) from E(t) for two assumed Poisson’s ratios. The NFL relaxation response is obtained for two strain rates: 1 × 10^8^/s and 1 × 10^9^/s. (**a**,**b**): 1 × 10^8^/s, (**c**,**d**): 1 × 10^9^/s.

We have performed a relaxation test on NFL. As NF sidearms dictate the sidearm spacing and eventually the axon diameter by orienting themselves as parallel filaments to MTs [30], we have decided to proceed by modeling the stress–strain response of NF sidearms. Figure 4 shows the pre-relaxation stretching and snapshots of the projection domain conformations at different timesteps during relaxation for NFL. Figure 5 shows the relaxation data (E(t) vs. time) for NFL; Table 1 shows the simplified viscoelastic parameters for relaxation modulus; Figure 6 shows the shear and bulk moduli obtained for two guessed Poisson’s ratios; and finally, Table 2 shows the shear and bulk moduli parameters. It is found that at lower strain rate, NFL acts as three-parameter material (according to our simplified model as defined in the Method section), while at higher strain rate, it acts as a two-parameter material.

## 4. Discussion

### 4.1. Strain Rate Dependence of Neurofilaments

The high strain rate behavior of NF is important from the traumatic brain injury (TBI) scenario, and their mechano-chemical behavior under the application of extreme strain is important to obtain insight regarding pathological phenomena. It is likely that their highly stretchable attribute is dictated by the intrinsic disorder in the sidearm region [14].

The NF behavior can also be explained from the potential effect of several parameters, such as conformational properties [20,21], charged state of sidearms network [23], ionic strength, and pH [69], which are highly pronounced in the normal (or dephosphorylated) state, and strong NF network formation due to significant interaction between charged network portions.

Studying NF behavior and quantifying their attributes against filament length is also important [24,31,32], as the previous studies were not able to confirm their relative importance from the isoform outlook. While some studies have shown that due to having the largest disordered tail region in the structure, NFH can dictate the behavior of the overall NF network, conflicting evidence has shown that NFH cannot unfold fully in phosphorylated state, and therefore, other isoforms may also play an instrumental role to regulate NF network response [51,52,53]. Furthermore, in a dynamic environment, while there are multiple biochemical parameters such as posttranslational modification and activation of multiple sites due to specific attacks on targeted residues, only phosphorylation in the sidearm region should not be overemphasized, as genetic studies have shown that wild-type and phosphorylation incompetent sidearms show no remarkable difference [29]. Earlier experiments substantiated that phosphorylation effect enhances the extensibility of the sidearms [70], and therefore, it is important to dictate the axonal diameter [25,26]. Multiple studies have further emphasized the importance of phosphorylation, as it is directly related to altering the charged state of the residues involved, and NF disassembly can be attributed to phosphorylation level. Therefore, a potential future direction is to incorporate sidearm phosphorylation to observe mechanical behavior in the presence of biochemical parameters.

Additionally, Atomic force microscopy (AFM) studies on NF have substantiated the presence of un-foldable regions in the sidearms, which explains the behavior of NF filaments to some extent in the current study [71]. Another important parameter is the charge distribution in the sidearm region, which suggests more anomaly in the case of longer filament [24,31,32]. The sidearm region in NF contains mostly negative, but few positively charged regions, and that decidedly alters the interaction in a one-to-one NF interaction scenario. Furthermore, the presence of a hydrophobic surface on the tail region adds further dynamic aspects while observing the phenomena from a biochemical perspective [15]. While considering NF accumulation, our conclusion is that a combination or mixture of NF-L, NF-M, and NF-H will provide comprehensive insight into their aggregation characteristics.

In short, the parameters that can be attributed to the unique aspects of mechanical behavior of NF are the charged portions, filament length, and intrinsic disorder.

### 4.2. Viscoelastic Behavior of Neurofilaments

In this study, we have performed relaxation on the sidearm region of NF-L, prior to which high strain rate stretching was performed on the filaments. Stretching at high strain rate provides two-fold conveniences: it allows us to proceed with the assumption that the loading region response can be ignored without losing a significant amount of accuracy in modeling the viscoelastic characteristics [48,72,73], and it facilitates mimicking a TBI scenario, where high deformation is observed in sub-axonal components [74].

NF contains several un-foldable regions of study [71], which cannot contribute to the stretching during the pre-relaxation. Therefore, the particular response can be attributed to a combination of high strain rate, disordered portion in the sidearm region, and the presence of un-foldable regions in the structure.

Lastly, to obtain a more comprehensive insight into the viscoelastic behavior of NF, two approaches can be taken. First, quantification of the response under slower and faster strain rate, without going into the failure region. Second, observing the viscoelastic response from a physical chemistry point of view by including effects like posttranslational modification as phosphorylation, as this phenomenon has been hypothesized to dictate functionality of NF [75,76,77]. In the near future, we intend to address these two aspects. In addition, this study provides particular insight into the time-dependent behavior of the most vulnerable regions of an important axonal cytoskeletal component in TBI scenario.

## 5. Conclusions and Prospect

In this study, the objective was to determine the mechanical behavior of NF, one of the constituents of axonal cytoskeleton. Therefore, we have computationally determined the mechanical behavior of the main isoforms of NFs by applying high strain rate relevant to the TBI scenario. Furthermore, we have attempted to develop simplified viscoelastic models for NFs using MD simulation. The major findings can be summarized as below:

NF contains multiple folding at unstretched state, while it unfolds to a great extent under the application of strain rate. The unfolding is smoother at smaller filament lengths (smaller isoforms, such as NF-L), while it shows highly unpredictable behavior for larger filament lengths (large isoforms, such as NF-H). The unique aspects of NF mechanical behavior can be attributed to the charged portions, intrinsic disorder in the sidearm, and filament length.

At lower strain rate, NF-L acts as a three-parameter material, but at higher strain rate, it corresponds to a two-parameter material. Such a response can be attributed to stretching time and stretched state at relaxation. A similar scheme can be extended to other filamentous subcellular structures, and SMD simulation can be a particularly useful tool to perform viscoelastic computations on such biomolecules.

The prospect of this study is to facilitate a realistic bottom-up computational modeling of axon. This requires insights regarding mechanical behavior of individual cytoskeletal components of an axon, specifically at high strain rate scenario. Therefore, this study provides insights into the mechanical behavior of NFs at strain rates relevant to TBI. In injury biomechanics area and especially in multiscale brain injury studies, these findings will play an instrumental role in determining damage criteria at the sub-axonal level and enhance the existing models of axons by providing insight regarding the mechanical behavior of axonal cytoskeletal components.

This study particularly sheds light on the sub-axonal level response of axonal cytoskeletal components of a neuron in TBI scenario, where nanoscale injury propagates (and the likely results are axonal damage and deformation of axonal cytoskeletal components such as MT instability, tau unfolding and stretching, tau-MT separation, NF unfolding, etc.) due to macroscale impact (head injury). To obtain more comprehensive insight into such axonal cytoskeletal components, we intend to perform studies incorporating physical chemistry parameters in future. As this study provides critical insight into the time-dependent response for an important axonal cytoskeletal component, it facilitates paving the pathway for an all-component-inclusive, realistic, bottom-up computational model for axons. In other words, this study will play a significant role in the enhancement of existing axon models that account for viscoelastic response of the cytoskeletal components and predict sub-axonal behavior in extreme strain rate conditions, which will contribute significantly to TBI research.

## Figures and Tables

**Table 1:** Simplified Model Parameters from NFL Relaxation Test.

Strain Rate, 1/s	1 × 10^8^	1 × 10^9^
E_1_, GPa	0.103	0
E_2_, GPa	0.827	2
τ, ps	87.866	100

**Table 2:** Relaxation Shear Modulus and Bulk Modulus Parameters for the Simplified Model of NFL.

Poisson’s Ratio, ν	0.33		0.48	
Strain Rate, 1/s	1 × 10^8^	1 × 10^9^	1 × 10^8^	1 × 10^9^
G_1_, GPa	0.039	0	0.035	0
G_2_, GPa	0.314	0.76	0.281	0.68
τ (for G), ps	90.909	100	90.909	100
K_1_, GPa	0.1	0	0.858	0
K_2_, GPa	0.81	1.96	6.889	16.66
τ (for K), ps	90.909	100	90.909	100

## Data Availability

The simulation data are available from the corresponding author upon reasonable request.

## Appendix A

Amino Acid Sequences of NF Isoforms (All are obtained from UniProt [72])

NF-L (Source: UniProt Database (UniProtKB-P07196 (NFL_HUMAN)))

MSSFSYEPYYSTSYKRRYVETPRVHISSVRSGYSTARSAYSSYSAPVSSSLSVRRSYSSS
SGSLMPSLENLDLSQVAAISNDLKSIRTQEKAQLQDLNDRFASFIERVHELEQQNKVLEA
ELLVLRQKHSEPSRFRALYEQEIRDLRLAAEDATNEKQALQGEREGLEETLRNLQARYEE
EVLSREDAEGRLMEARKGADEAALARAELEKRIDSLMDEISFLKKVHEEEIAELQAQIQY
AQISVEMDVTKPDLSAALKDIRAQYEKLAAKNMQNAEEWFKSRFTVLTESAAKNTDAVRA
AKDEVSESRRLLKAKTLEIEACRGMNEALEKQLQELEDKQNADISAMQDTINKLENELRT
TKSEMARYLKEYQDLLNVKMALDIEIAAYRKLLEGEETRLSFTSVGSITSGYSQSSQVFG
RSAYGGLQTSSYLMSTRSFPSYYTSHVQEEQIEVEETIEAAKAEEAKDEPPSEGEAEEEE
KDKEEAEEEEAAEEEEAAKEESEEAKEEEEGGEGEEGEETKEAEEEEKKVEGAGEEQAAK
KKD
		

NF-M (Source: UniProt Database (UniProtKB-P07197 (NFM_HUMAN)))

MSYTLDSLGNPSAYRRVTETRSSFSRVSGSPSSGFRSQSWSRGSPSTVSSSYKRSMLAPR
LAYSSAMLSSAESSLDFSQSSSLLNGGSGPGGDYKLSRSNEKEQLQGLNDRFAGYIEKVH
YLEQQNKEIEAEIQALRQKQASHAQLGDAYDQEIRELRATLEMVNHEKAQVQLDSDHLEE
DIHRLKERFEEEARLRDDTEAAIRALRKDIEEASLVKVELDKKVQSLQDEVAFLRSNHEE
EVADLLAQIQASHITVERKDYLKTDISTALKEIRSQLESHSDQNMHQAEEWFKCRYAKLT
EAAEQNKEAIRSAKEEIAEYRRQLQSKSIELESVRGTKESLERQLSDIEERHNHDLSSYQ
DTIQQLENELRGTKWEMARHLREYQDLLNVKMALDIEIAAYRKLLEGEETRFSTFAGSIT
GPLYTHRPPITISSKIQKPKVEAPKLKVQHKFVEEIIEETKVEDEKSEMEEALTAITEEL
AVSMKEEKKEAAEEKEEEPEAEEEEVAAKKSPVKATAPEVKEEEGEKEEEEGQEEEEEED
EGAKSDQAEEGGSEKEGSSEKEEGEQEEGETEAEAEGEEAEAKEEKKVEEKSEEVATKEE
LVADAKVEKPEKAKSPVPKSPVEEKGKSPVPKSPVEEKGKSPVPKSPVEEKGKSPVPKSP
VEEKGKSPVSKSPVEEKAKSPVPKSPVEEAKSKAEVGKGEQKEEEEKEVKEAPKEEKVEK
KEEKPKDVPEKKKAESPVKEEAVAEVVTITKSVKVHLEKETKEEGKPLQQEKEKEKAGGE
GGSEEEGSDKGAKGSRKEDIAVNGEVEGKEEVEQETKEKGSGREEEKGVVTNGLDLSPAD
EKKGGDKSEEKVVVTKTVEKITSEGGDGATKYITKSVTVTQKVEEHEETFEEKLVSTKKV
EKVTSHAIVKEVTQSD
		

NF-H (Source: UniProt Database (UniProtKB-P12036 (NFH_HUMAN)))

MMSFGGADALLGAPFAPLHGGGSLHYALARKGGAGGTRSAAGSSSGFHSWTRTSVSSVSA
SPSRFRGAGAASSTDSLDTLSNGPEGCMVAVATSRSEKEQLQALNDRFAGYIDKVRQLEA
HNRSLEGEAAALRQQQAGRSAMGELYEREVREMRGAVLRLGAARGQLRLEQEHLLEDIAH
VRQRLDDEARQREEAEAAARALARFAQEAEAARVDLQKKAQALQEECGYLRRHHQEEVGE
LLGQIQGSGAAQAQMQAETRDALKCDVTSALREIRAQLEGHAVQSTLQSEEWFRVRLDRL
SEAAKVNTDAMRSAQEEITEYRRQLQARTTELEALKSTKDSLERQRSELEDRHQADIASY
QEAIQQLDAELRNTKWEMAAQLREYQDLLNVKMALDIEIAAYRKLLEGEECRIGFGPIPF
SLPEGLPKIPSVSTHIKVKSEEKIKVVEKSEKETVIVEEQTEETQVTEEVTEEEEKEAKE
EEGKEEEGGEEEEAEGGEEETKSPPAEEAASPEKEAKSPVKEEAKSPAEAKSPEKEEAKS
PAEVKSPEKAKSPAKEEAKSPPEAKSPEKEEAKSPAEVKSPEKAKSPAKEEAKSPAEAKS
PEKAKSPVKEEAKSPAEAKSPVKEEAKSPAEVKSPEKAKSPTKEEAKSPEKAKSPEKAKS
PEKEEAKSPEKAKSPVKAEAKSPEKAKSPVKAEAKSPEKAKSPVKEEAKSPEKAKSPVKE
EAKSPEKAKSPVKEEAKTPEKAKSPVKEEAKSPEKAKSPEKAKTLDVKSPEAKTPAKEEA
RSPADKFPEKAKSPVKEEVKSPEKAKSPLKEDAKAPEKEIPKKEEVKSPVKEEEKPQEVK
VKEPPKKAEEEKAPATPKTEEKKDSKKEEAPKKEAPKPKVEEKKEPAVEKPKESKVEAKK
EEAEDKKKVPTPEKEAPAKVEVKEDAKPKEKTEVAKKEPDDAKAKEPSKPAEKKEAAPEK
KDTKEEKAKKPEEKPKTEAKAKEDDKTLSKEPSKPKAEKAEKSSSTDQKDSKPPEKATED
KAAKGK
		

## Appendix B

Simulation Setup

**Table A1:** Simulation Setup in Terms of Box Size, Number of Atoms, and Filament Length.

	NF-L	NF-H
Number of atoms in NF	8486	15,882
Number of atoms in the simulation box (including water and ions)	567,706	757,130
Simulation box size (nm × nm × nm)	40 × 18 × 10	40 × 20 × 10
Approximate filament length in nm (N.B. the end-to-end distance of the filament, which is not in a straight conformation, is being mentioned here as “length” to provide an idea of the protein placement in the simulation box)	17.3	17.3
